# Functional and morphological changes in hypoplasic posterior fossa

**DOI:** 10.1007/s00381-021-05193-w

**Published:** 2021-06-25

**Authors:** Federico Bianchi, Alberto Benato, Paolo Frassanito, Gianpiero Tamburrini, Luca Massimi

**Affiliations:** 1 Neurochirurgia Infantile, Fondazione Policlinico Universitario A. Gemelli IRCCS, Rome, Italy; 2 Università Cattolica del Sacro Cuore, Rome, Italy

**Keywords:** Posterior cranial fossa, Craniosynostosis, Chiari I malformation, Hydrocephalus, Achondroplasia, Precision medicine

## Abstract

**Background:**

The knowledge of the development and the anatomy of the posterior cranial fossa (PCF) is crucial to define the occurrence and the prognosis of diseases where the surface and/or the volume of PCF is reduced, as several forms of craniosynostosis or Chiari type I malformation (CIM). To understand the functional and morphological changes resulting from such a hypoplasia is mandatory for their correct management. The purpose of this article is to review the pertinent literature to provide an update on this topic.

**Methods:**

The related and most recent literature addressing the issue of the changes in hypoplasic PCF has been reviewed with particular interest in the studies focusing on the PCF characteristics in craniosynostosis, CIM, and achondroplasia.

**Results and conclusions:**

In craniosynostoses, namely, the syndromic ones, PCF shows different degrees of hypoplasia, according to the different pattern and timing of early suture fusion. Several factors concur to PCF hypoplasia and contribute to the resulting problems (CIM, hydrocephalus), as the fusion of the major and minor sutures of the lambdoid arch, the involvement of the basal synchondroses, and the occlusion of the jugular foramina. The combination of these factors explains the variety of the clinical and radiological phenotypes. In primary CIM, the matter is complicated by the evidence that, in spite of impaired PCF 2D measurements and theories on the mesodermal defect, the PCF volumetry is often comparable to healthy subjects. CIM is revealed by the overcrowding of the foramen magnum that is the result of a cranio-cerebral disproportion (altered PCF brain volume/PCF total volume). Sometimes, this disproportion is evident and can be demonstrated (basilar invagination, real PCF hypoplasia); sometimes, it is not. Some recent genetic observations would suggest that CIM is the result of an excessive growth of the neural tissue rather than a reduced growth of PCF bones. Finally, in achondroplasia, both macrocephaly and reduced 2D and 3D values of PCF occur. Some aspects of this disease remain partially obscure, as the rare incidence of hydrocephalus and syringomyelia and the common occurrence of asymptomatic upper cervical spinal cord damage. On the other hand, the low rate of CIM could be explained on the basis of the reduced area of the foramen magnum, which would prevent the hindbrain herniation.

## Introduction

Posterior cranial fossa (PCF) represents a common ground for pediatric neurosurgeons, its development and morphology being crucial in several pathological conditions. Therefore, independently from the type of disease, a comprehensive knowledge of its morpho-functional characteristics is mandatory to define properly the treatment strategy.

Among the possible findings concerning PCF, the present “focus session” mainly addresses the hypoplasia, in particular that resulting from craniosynostosis.

Phylogenetical studies underlined how the origin of PCF, as we know today, derived from an increase in supratentorial brain volume combined with bipedal stance leading to a need in weight redistribution. A central role in such a redistribution is performed by the prominence of the human tentorium that has the role of bearing the cerebral weight as well as distributing this load to the osseous skull. Such a need led to the fetal tentorium downward rotation in response to the disproportionate growth of the human cerebrum compared with the cerebellum [1].

The osseous counterpart of the tentorial rotation is explained by a two-timed mechanism of PCF growth. The first moment occurs during early childhood, while the latter happens during puberty. During the childhood, PCF grows in length mainly alongside the intra-occipital, petro-occipital, and spheno-occipital synchondrosis, while, during the puberty, it enlarges following mainly the spheno-occipital growth [2]. This different timing in growth is related to an initial absence of ossification in the petro-occipital, the occipito-mastoid, and the spheno-occipital synchondroses. The aforementioned mechanism is different between males and females, the rapid growth phase being shorter and faster in girls [3, 4].

Only a few studies in the literature are focused on the normal volume of PCF or on its measures in healthy children. Coll and colleagues observed a bigger PCF in boys compared with girls at the endo of the development, as results of a smaller PCF size in the girls at birth [3]. Rehder et al. measured the mean tentorial angle (the angle between tentorium and a line from the internal occipital protuberance to tuberculum sellae) in healthy children, which was 42.38 degrees, and the mean occipital angle (the angle between the tentorium and a line from internal occipital protuberance to opisthion), which was 90.58 degrees [5, 6].

## Impact of PCF changes on craniosynostosis

### Chiari I malformation

**Fig. 1:**
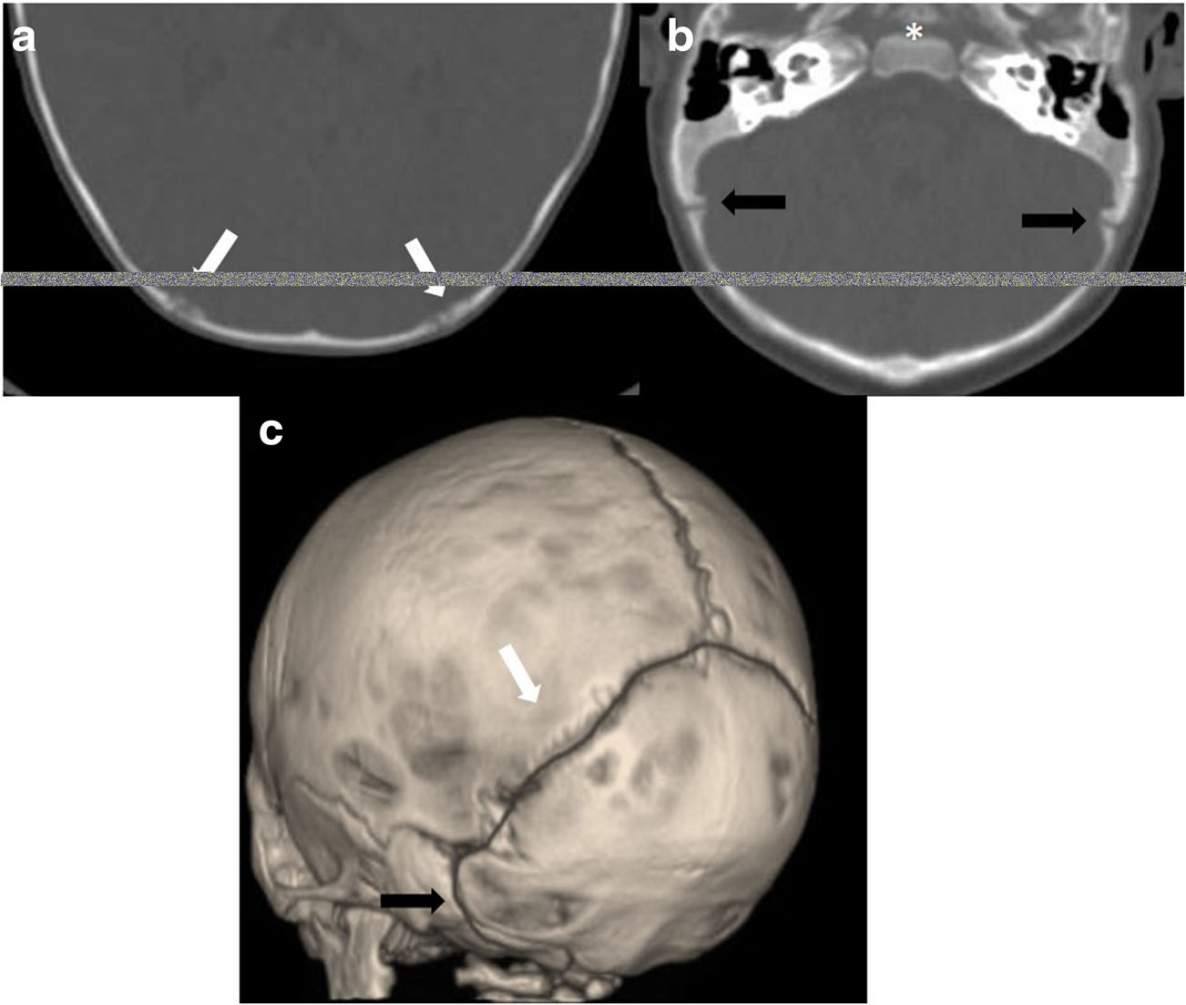
The lambdoid arch on 2D (**a**, **b**) and 3D CT scan (**c**) of the skull. Both the lambdoid (white arrows) and the occipito-mastoid sutures (black arrows) as well as the spheno-occipital synchondrosis (asterisk) can be appreciated

**Fig. 2:**
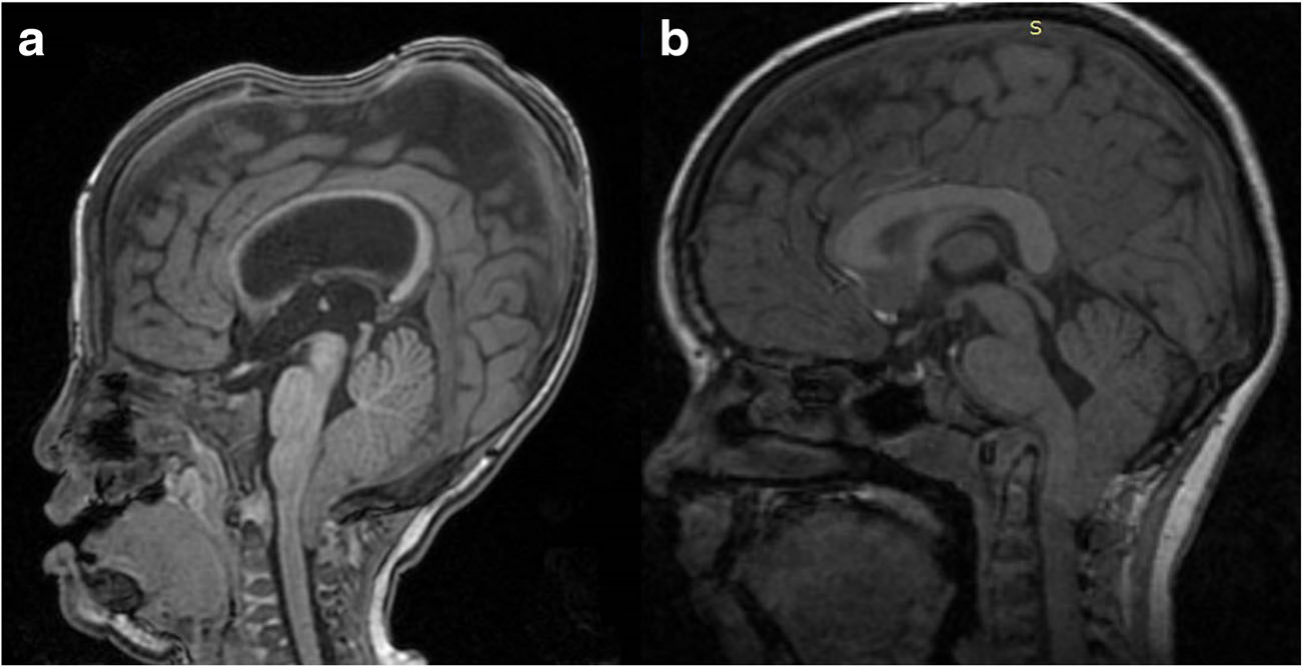
Sagittal MRI showing CIM as a result of two different conditions: **a** Crouzon syndrome (18-month-old girl), note the severe FCP hypoplasia, the marked verticalization of the tentorium, the upward and downward displacement of the cerebellum, and the sellar deformation of the neurocranium, **b** primary CIM associated with basilar invagination (5-year-old boy), the PCF hypoplasia is moderate but a significant kinging of the bulbo-pontine junction is present. In both cases, the foramen magnum is large enough to allow the hindbrain herniation

Children with craniosynostosis showing a premature fusion of the posterior arch sutures (Fig. 1) are prone to develop PCF hypoplasia, often with secondary Chiari I malformation (CIM). CIM and the possibly resulting syringomyelia are the key issue arising from the functional and morphological changes of hypoplasic PCF in craniosynostosis, all the main PCF components (neural tissue, cerebrospinal fluid (CSF), vessels, bone) being involved in their genesis. Therefore, several hypotheses have been proposed as far as the pathophysiological mechanism leading to the association of these two conditions is concerned. According to Cinalli and colleagues, the downward herniation of the cerebellar tonsils would result from the disproportion between the neural (normal or enlarged) and the bony component (narrowed) of the PCF [2]. The premature fusion of multiple sutures, especially those belonging to the posterior cranial arch, is considered the main cause of such a cephalo-cranial disproportion (Fig. 2a). This hypothesis is empowered by the progressive appearance of CIM in craniosynostotic children depending on the different timing of closure of the sutures: the earlier the premature fusion of these sutures (e.g., Crouzon syndrome), the higher the risk of CIM (such a risk is actually lower when the sutures close later, as in Apert syndrome). The authors also pointed some findings, such as the venous engorgement due to the occlusion of the jugular foramina and the associated hydrocephalus, as possible factors increasing the cranio-cerebral disproportion (brain turgor). In syndromic children, this brain turgor is exacerbated by the chronic CO2 retention due to the upper airway’s obstruction [7]. On these grounds, Coll and colleagues consider at least three different theories together behind the presence of CIM in craniosynostosis: the constrictive, the venous, and the foramen magnum stenosis theories. In summary, the presence of a small PCF would result in a mechanical increase in resistance to the CSF flow, which is complicated by the venous hypertension determined by the jugular foramina stenosis and by the foramen magnum stenosis with subsequent subarachnoid CSF blockage [8, 9]. As mentioned, the progression of the conflict between neural and skull growth becomes critical when the posterior arch (in particular, the lambdoid sutures) closes within the first 2 years of life, during this time the cerebellar volume being increased if compared with the forebrain. The timeline suggested by the authors in CIM development is the following:

1. Progressive fusion of the lambdoid suture with secondary skull base alteration
2. Stenosis of the jugular foramina
3. Hypoplasic PCF growth
4. Cerebellar herniation
5. Venous hypertension
6. Crowding of the posterior fossa;
7. CSF hydrodynamics alteration

In addition, a low attachment of the tentorium can reduce the size of the posterior fossa, further feeding the vicious circle [2]. The role of the stenosis of the foramen magnum is discussed below.

The aforementioned authors also reported on some genetic findings related to the PCF hypoplasia in syndromic craniosynostosis. Actually, *FGFR2* mutations involving exons 3a and 3c occur more frequently in patients developing CIM [2, 3, 10]. Among the *FGFR2* patients, as anticipated, the phenotypic alteration can greatly change the PCF dimension alongside the different syndromes. Children with Apert syndrome have a larger than normal basiocciput, while those affected by Crouzon syndrome tend to show one smaller than normal and expanding mainly along the supero-inferior axis rather than the antero-posterior one. As a result, the PCF volume in Apert patients is often normal or even increased, while it is more or less severely hypoplasic in Crouzon patients [2, 11].

### Main PCF changes

A further field of research is represented by the analysis of the size of PCF in craniosynostosis-related CIM. To assess that, some authors used bi-dimensional parameters, like PCF cephalic index, where the skull length was measured from the posterior edge of clivus to the inner table of the occiput and the skull width between the orifices of the right and left internal auditory canals; tentorial angle that is the angle between the tentorium and a line running from the internal occipital protuberance to the tuberculum sellae; and the occipital angle that is the angle between the tentorium and a line from internal occipital protuberance to opisthion [2, 5, 12]. According to these studies, children affected by syndromic and bi-coronal synostosis are more prone to develop CIM than those affected by single suture synostoses because of the brachycephalic skull and the steep tentorium (high PCF cephalic index and large tentorial and occipital angle). On the contrary, in scaphocephalic patients, although present, the risk to develop CIM is inferior due to the higher incidence of flat tentorium [2, 5, 12]. More in details, PCF cephalic index was 0.62 in sagittal synostosis, 0.74 in metopic synostosis, 0.93 in bi-coronal synostosis, 0.79 in Pfeiffer syndrome, 0.83 in Crouzon syndrome, and 0.78 in healthy children. The mean tentorial angle and mean occipital angle were, respectively, 45.48 and 96.68 in syndromic synostoses, 39.78 and 87.08 in bi-coronal synostosis, 34.0 and 75.08 in sagittal synostosis, 39.78 and 87.08 in metopic synostosis, and 42.98 and 88.38 in controls.

**Fig. 3:**
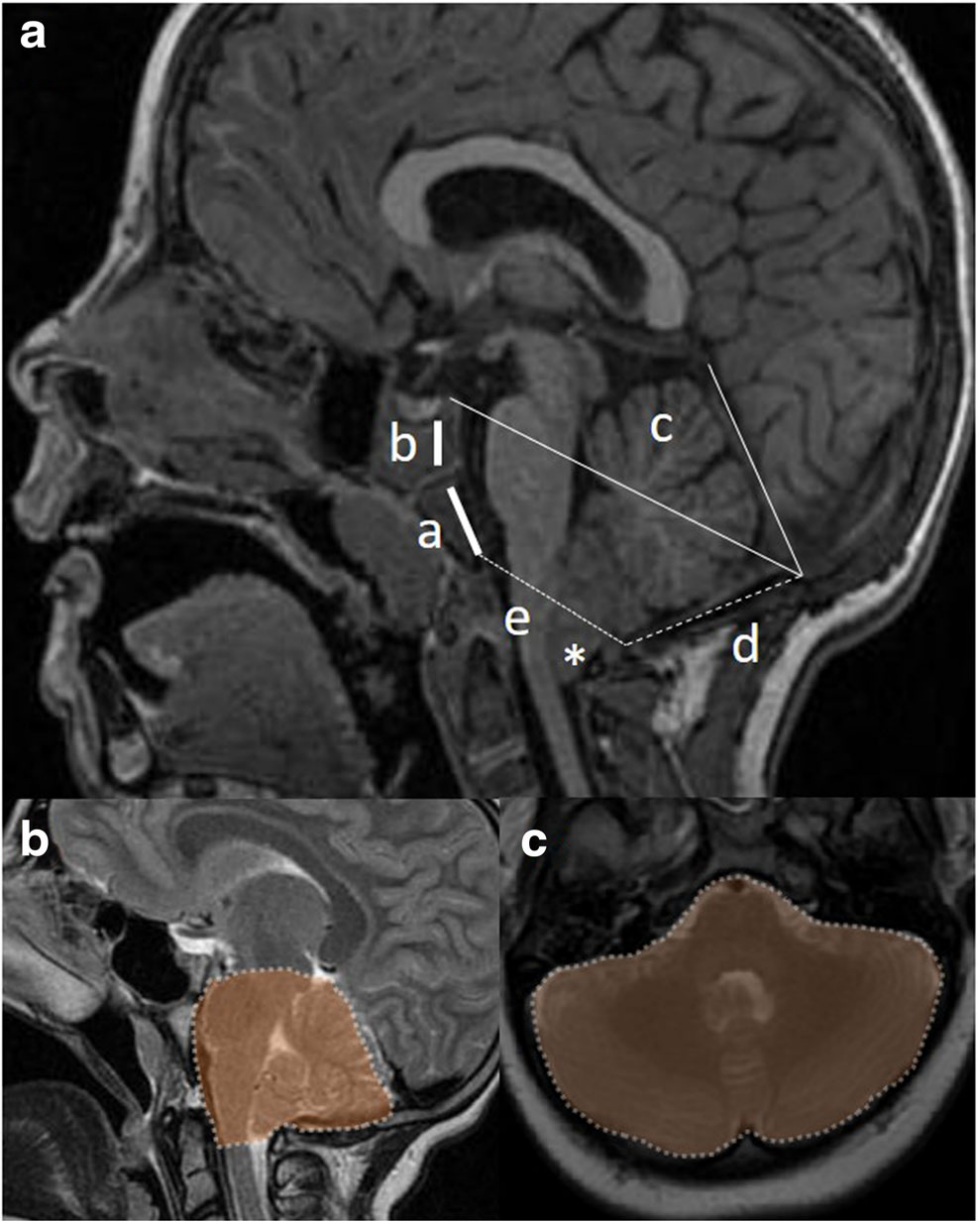
Main bi-dimensional PCF measurements on MRI (A): clivus basi-occiput (a), clivus basi-sphenoid (b), tentorial angle (c), supra-occiput (d), basion-opisthion line, and (e) to estimate the tonsillar descent (asterisk). Sagittal (B) and axial MRI (C) showing the template for the PCF three-dimensional assessment

To complete this kind of measurements, other authors performed a volumetric analysis of PCF. For this purpose, Calandrelli et al. used several parameters, such as sutural pattern, 2D PCF morphometric analysis (basiocciput, exocciput, supraocciput, and meatus-opisthion lengths, antero-posterior and latero-lateral foramen magnum diameter, tentorial angle, jugular foramina areas) and 3D PCF volumetric analysis (PCF volume, PCF brain volume, PCF volume/PCF brain volume ratio, cerebellum and CSF volume) on computed tomography (CT) and magnetic resonance imaging (MRI) of children with multisutural synostoses versus controls (Fig. 3) [13]. According to their results, all children (both syndromic and non-syndromic) showed the involvement of the lambdoid arch, 40% of them with involvement only of the lambdoid sutures (major sutures), 40% with involvement only of the minor sutures (occipito-mastoid and occipito-petrosal sutures), and the remaining 20% with involvement of both patterns. In case of major sutures involvement, all the measurements were comparable with the controls except for the exocciput lengths and the antero-posterior diameter of the foramen magnum (significantly reduced) and the tentorial angle (significantly increased). The measurements were similar to controls also in case of minor sutures involvement, apart from a significant increase of the tentorial angle and a significant decrease of the meatus-opisthion lengths, the diameters of the foramen magnum (both antero-posterior and latero-lateral), and the jugular foramina area. Subsequently, in case of isolated closure of minor lambdoid arch sutures, the PCF hypoplasia mainly depends on the reduced length of the two hemi-fossae: these findings allow the cerebellum to grow rostrally, and, together with a complete reduction of the area of the foramen magnum, they explain the absence of CIM in this subset of patients (CIM may occur later on, when the tentorium reaches its maximum slope). On the other hand, in case of major sutures closure, PCF is hypoplasic because of a reduced exocciput length, so that the cerebellum is forced to growth both rostrally and caudally and, thanks to a normal antero-posterior diameter of the foramen magnum, early CIM occurs (80% of cases). These observations would explain also why CIM is most likely to occur with isolated major suture involvement (e.g., asymmetrical, ipsilateral tonsillar herniation in lambdoid synostosis) than in minor one, even though both conditions are characterized by PCF hypoplasia.

By applying the same method to sagittal synostosis, the same authors identified the mismatch between the reduced compliance of the skull base and the compensatory increase of the supratentorial cranial volume as the possible cause leading to hindbrain descent in severe sagittal synostosis compared with mild cases and controls [14]. More in details, anterior, middle, and posterior cranial fossae were symmetric as well as the anterior and middle length of the skull base were increased in mild, moderate, and severe sagittal synostosis, but the compensatory enlargement of the anterior fossae was found to be significant only in mild and moderate groups. The lengths of the posterior fossae were comparable in all groups. These observations suggest that the spontaneous calvarial remodeling drives the anterior and middle skull base enlargement probably because the central section of the sagittal suture is the first to fuse; in the severe group, the minor elongation of the skull base could be related to the earlier timing of sagittal suture synostosis. Under a volumetric point of view, this corresponds to a major compensatory intracranial volume in mild forms compared with the severe ones. Concerning the infratentorial volume, a normal PCF volume was found in every group, but, in moderate and severe groups, the ratio between PCF volume and PCF brain volume and the infratentorial CSF volume were significantly reduced, thus pointing out a “brain constriction” that could lead to CIM in the more severe cases.

The study by Leikola et al., who compared craniosynostotic subjects with and without CIM, showed that children with CIM have an antero-posterior PCF length significantly shorter than those without CIM (33.4 mm vs 36.7 mm) [15]. Similarly, the cross-sectional area was 654.1 versus 764.9 mm^2^, respectively, while there was not a significant difference in the mean width between CIM children (28.1 mm) and no-CIM children (29.9 mm). In CIM children, the foramen magnum cross-sectional area was about 88% of no-CIM children.

The previous studies give reason of the occurrence of CIM even in not syndromic synostosis, which is as high as 60% in lambdoid and 5% in sagittal craniosynostosis [16, 17]. They also explain why CIM is found even in apparently less “predisposing” synostosis, like unicoronal craniosynostosis (sometimes more frequently than in sagittal synostosis) [18], because of the secondary anterior attraction and shortening of one posterior hemi-fossa. The combined observations done in single suture synostoses justify the high incidence of CIM in multisutural, not syndromic synostoses, such as Mercedes syndrome (about 60%) and oxycephaly (80–100%) [19, 20].

It is worth noting that linear or superficial PCF measurements might not be accurate enough in assessing the risk of CIM occurrence in the craniosynostosis population. According to the Rijken and coworkers’ study, indeed, children with Muenke syndrome showed both a bigger cerebellar (CV) and PCF volume (PFV) than those with Apert syndrome, in which only PCF volume resulted larger than normal [21]. These findings, coupled with the higher frequency of mega cisterna magna in Apert syndrome, explain the significantly lower CV/PFV ratio in these subjects (and, so, the lower risk of CIM). The author proposed the CV/PFV ratio as a reliable indicator of PCF crowding because, even in case of significant PCF hypoplasia, CIM does not occur if also the cerebellum is hypoplasic. Nonetheless, CV/PFV ratio in CIM-craniosynostosis patients partially overlaps that of the controls, ranging between 0.73 and 0.81 in affected children and from 0.69 to 0.81 in healthy subjects [21].

As mentioned, also the vascular modification plays a crucial role in CIM development. A clear explanation on the cause of the venous obstruction is still missing. Some studies suggest an error in the primary meningeal or osseous development, while others blame a condition of chronic PCF hypertension [9]. Small or stenotic jugular foramina, with abnormal course of the jugular veins and extensive trans-osseous venous collaterals (parietal, occipital, and mastoid emissaries; marginal, occipital, and cavernous sinuses; the ophthalmic veins; and the external jugular vein) are the main finding in craniosynostosis. The more these findings are present, the higher the risk of intracranial hypertension. Syndromic patients are more prone to develop the aforementioned venous anomalies because of the high incidence of narrowed jugular foramina, Crouzon, and Pfeiffer patients showing more extensive venous collaterals than Apert and Saethre-Chotzen ones [9].

### Hydrocephalus

Hydrocephalus complicates the clinical course of syndromic craniosynostosis in 12–15% of cases and about 90% of hydrocephalic children with syndromic synostosis are affected by CIM too [22]. The strict correlation among PCF bony (hypoplasia, basilar invagination), neural (foramen magnum overcrowding/CIM, compression of the IV ventricle), vascular (jugular foramina stenosis), and CSF changes (impaired circulation) allow to explain the association between hydrocephalus and syndromic synostoses. The multifactorial genesis of this peculiar type of hydrocephalus shares some points with the timeline of CIM and can be summarized as follows [2, 23]: (1) PCF hypoplasia causes a “minor” impairment in the CSF circulation because of the direct compression on the IV ventricle. (2) PCF hypoplasia causes a “major” impairment of the CSF circulation (indirectly) by preventing the CSF flow at level of the foramen magnum. This would be the result of the cephalo-cranial disproportion leading to the overcrowding of the foramen magnum. The raised ICP resulting from the impaired CSF circulation worsens the foramen magnum overcrowding (CIM), feeding a vicious circle. (3) The stenosis of the jugular foramina favors the hydrocephalus by increasing the cephalo-cranial disproportion (cerebellar edema due to the venous engorgement), by increasing the raised intracranial pressure (upward venous hypertension) and by decreasing the CSF re-adsorption. (4) The stenosis of the jugular foramina is able to induce hydrocephalus, with the same mechanisms as above, even if the PCF hypoplasia is missing (absent early fusion of both major and minor posterior arch sutures).

## Impact of PCF changes on primary Chiari I malformation

### Anatomical features of PCF in CIM

CIM is defined by PCF hypoplasia, herniation of the cerebellar tonsils across McRae’s line and reduction of PCF subarachnoid spaces. The cutoff of the tonsillar herniation ranges between 3 and 5 mm (criteria vary according to the source), but, today, it is widely accepted that such a cutoff does not have a significant clinical relevance, as the entity of tonsillar herniation does not strictly correlate with the symptoms [24, 25]. Other than the definition, several efforts are currently produced to assess the controversial clinical symptoms and to provide some guidelines for the management. On the other hand, several investigations have been carried out regarding CIM pathophysiology, aiming at identifying the genesis of the disease and the distinctive features of symptomatic and asymptomatic cases and exploring the pathophysiologic correlation between the CIM-related PCF changes and their consequences (syringomyelia and/or hydrocephalus). In particular, most of the studies are focused on PCF anatomy and CSF hydrodynamics at the level of the foramen magnum, which point CIM as a complex phenomenon originating from the dynamic interplay between the developing bone of PCF and its neural, vascular, and CSF contents.

Several morphometric studies have been realized so far, highlighting significant differences in PCF parameters between CIM patients and the general population. Smaller PCF volume [26], shorter clival length, shorter PCF length and height, and platybasia [27, 28] have been recognized as distinctive features in CIM patients. The origin of these abnormalities is thought to lie in the underdevelopment of the occipital somite that gives origin to the mature occipital bone, as evidenced in the seminal studies conducted by Marin-Padilla and colleagues [29]. According to these authors, an insufficient growth of the para-axial mesoderm, when occurring after closure of the neural folds, would lead to an abnormal development of the whole basichondrocranium, with consequences ranging from a “simply” small PCF to a severe kyphosis at the cranio-cervical junction with basilar invagination. This would cause a mismatch between posterior fossa dimensions and growing neural contents, in turn bringing herniation of PCF contents (especially the tonsils) through the foramen magnum. The pioneering study by Nishikawa and coworkers confirmed this hypothesis and put the basis for the future measurements of PCF in CIM patients [30]. The authors investigated 30 CIM adults and 50 controls by CT scan and MRI. Neuroimaging was used to define some linear bony parameters, as the basiocciput (axial length of the clivus measured from the top of the dorsum sellae to the basion), the supraocciput (distance between the internal occipital protuberance and the opisthion measured on midline cuts), and the exocciput (distance from the bottom of the occipital condyle to the top of the jugular tubercle at the level of the hypoglossal canal in antero-posterior cuts vertical to the orbitomeatal line); to measure the brain structures inside PCF (midbrain, pons, cerebellum, and medulla oblongata); and to calculate the PCF brain volume (brainstem and cerebellum) and the overall PCF volume (neural tissue and CSF spaces and bone). The ratio between PCF brain volume and total PCF volume was used as indicator of PCF overcrowding. As a result, (1) CIM patients showed a significant reduction of exocciput and supraocciput compared to controls, and a not significant reduction of basiocciput. (2) All these three parameters were strongly reduced in CIM patients harboring basilar invagination. (3) The tentorium was significantly more vertical in CIM patients than in controls. (4) There were no significant differences in the PCF brain and total volumes between CIM cases (with and without basilar invagination) and controls. (5) But there was a significant difference about the PCF brain volume/total volume ratio, thus indicating a PCF overcrowding in CIM patients (even more severe when dealing with those with basilar invagination). According to the authors’ conclusions, CIM would result from an underdeveloped occipital bone (possibly due to underdevelopment of the occipital somite originating from the paraxial mesoderm) which induces overcrowding in a PCF containing normal neural tissue. Basilar invagination worsens the hindbrain herniation due to the more severely underdeveloped occipital enchondrium (Fig. 2b). This data has been confirmed by several other studies [31–33]. However, it is worth noting that both linear and volumetric values in CIM patients may overlap those of healthy subjects, especially if they are matched with the demographic characteristics of the patients [34]. The studies carried out specifically on children confirmed that PCF volumes and ratio are often normal in CIM subjects, only children with syringomyelia showing a significant PCF overcrowding [35, 36]. In spite of that, and irrespectively of the presence of syringomyelia, the linear measurements of the bone landmarks can be abnormal compared with controls (larger angle formed by the crista galli-dorsum sellae-foramen magnum, larger angle formed by the two internal auditory meatuses and the foramen magnum, longer distance between the two internal auditory meatuses), thus indicating a mesodermal defect [37]. These figures demonstrate that the pathogenesis of CIM is probably multifactorial and still need to be clarified.

### Contribution by the genetics

In recent years, the neuroimaging morpho-volumetric studies have been more and more integrated by genetic studies. First, these investigations supported the hypothesis about a genetic origin of CIM, due to the familiar aggregation or clustering and the occurrence of CIM in some syndromes (e.g., Klippel-Feil syndrome, primary basilar impression, Goldenhar syndrome) [38]. Moreover, animal models and studies on some human hereditary skeletal diseases allowed to identify some molecular pathways involved in the skull growth and ossification (*FGF*, *Hedgehog*, *BMP*, *Wnt* pathways), which could contribute to explain a possible PCF mesoderm defect in CIM [39]. In this context, it is not surprising that the FGF signaling pathway is shared with craniosynostosis. Such a relationship was already pointed out several years ago by a genome-wide linkage study about 23 CIM families where a significant association to chromosomes 9 and 15 was found [40]. The 15q21.1-q22.3 region corresponds to the *fibrillin 1* gene, which is mutated in patients with Shprintzen-Goldberg syndrome (a syndrome with craniosynostosis and brain anomalies including CIM). More recently, the same type of analysis revealed a linkage to regions on chromosomes 8 and 12 harboring the genes of some growth differentiation factors (*GDF6* and *GDF3*) implicated in Klippel-Feil syndrome (associated with CIM in 3–5% of cases) [41].

As far as the topic of the present article is concerned, a relevant support is coming from the whole exome sequencing studies. By using this approach on 7 extended families (62 samples), Musolf et al. identified two loci, 1q43-44 and 12q23-24.11, possibly correlating with a small PCF in CIM patients [42]. The authors performed both linear (clivus, basiocciput, and supraocciput length) and volumetric measurements (PCF volume/supratentorial volume ratio) of their patients and matched them with the exome sequencing results. A small PCF correlated with *MYBPC1* gene (12q23.2), which encodes for proteins responsible of the striate muscle contraction and is involved in the genesis of arthrogryposis, and *AKT3* and *COX20* genes (1q43-44), implicated in microcephaly and cerebellar ataxia/muscle hypotonia, respectively. Although the causality of these genes has to be proved yet, this study suggests that CIM could arise from the action of different genes, the combination of which would explain the differences in the PCF morpho-volumetric analysis and the absence of a significant PCF hypoplasia in several CIM subjects compared to controls. Furthermore, a very recent multicenter study, based on the whole exome sequencing of 232 CIM families (668 patients), showed that chromodomain genes (namely, *CHD3* and *CHD8* genes) can contribute to CIM genesis [43]. These genes are implicated in macrocephaly (as it happens in zebrafishes), and macrocephaly (large head circumference without associated brain or CSF anomalies) was largely present in the families enrolled for this study. Accordingly, CIM would be the result of an excessive brain growth rather than a too small PCF.

### Syringomyelia

From a clinical point of view, the classical interpretation of the neuroimaging studies has been that foramen magnum overcrowding causes disturbances in CSF outflow from the cranial compartment, in turn potentially leading to syringomyelia and CIM clinical symptoms. However, even if all CIM patients share the described abnormalities (small PCF, hindbrain herniation, effacement of CSF spaces), only a subset of them develops syringomyelia. Foramen magnum overcrowding is thought to generate an intra-extracranial CSF pressure gradient which pulsates in line with the cardiac cycle. These abnormal pressure waves would cause progressive penetration of CSF into the perivascular spaces of the spinal cord [44]. The severity of crowding and tonsillar descent shows only a poor correlation with clinical symptoms [44–46]. In this context, one study specifically investigated the anatomical parameters that could help to distinguish between CIM with and without syringomyelia [47]. By evaluating 67 CIM patients, Yan et al. found that while the main PCF parameters (clival length, posterior fossa length and height, supraocciput length) did not differ significantly between CIM cases with and without syringomyelia, patients with a syrinx had a significantly smaller clival gradient (defined as the anterior angle between the clivus and McRae’s line). This represents a more pronounced platybasia (interestingly, clival gradient did not differ significantly between CIM patients without syringomyelia and normal controls). The authors proposed that, among the CSF turbulences and flow alterations occurring in CIM patients, the ones responsible for syrinx formation are those occurring in the front of the foramen magnum. Patients with more pronounced platybasia would be more affected due to the reduction in the pre-pontine and pre-bulbar cisternal space and consequent local turbulences in the anterior PCF compartment. This hypothesis appears to be corroborated by the inverse relationship between clival gradient and syrinx severity. The main limitation of the study is that the greatest part of the involved patients had only mild or absent clinical symptoms, thus leaving unexplored the true clinical significance of the described observations.

These findings create an interesting link with the studies on CSF hydrodynamics that have been performed with direct measurements or through indirect CSF flow estimates (dedicated MRI cine sequences) [44]. In particular, some studies seem to confirm that in CIM the most relevant alterations of CSF flow occur in the anterior portion of the foramen magnum [44]. This also suggests that the difference between symptomatic and asymptomatic patients could lay in focal turbulences of CSF flow, thus questioning the appropriateness of considering only global CSF movements across the foramen magnum. Iskandar and colleagues noticed that it is no longer sufficient to rely only on mean flow parameters to correctly define CSF flow alteration in PCF of CIM patients (with and without syringomyelia). In fact, it seems that the more relevant alteration in CSF dynamics interest the anterior portion of the foramen magnum and, in particular, some small sub-regions of the anterior quadrants, either unilaterally or bilaterally. When these regions show higher CSF velocity flows, there is a higher risk to develop syringomyelia. This finding appears to be statistically significant and to correlate with postoperative results. Actually, if there is a postoperative decrease in CSF velocity flow in the aforementioned areas, syringomyelia is more likely to decrease or disappear [44].

### Hydrocephalus

Although the first etiologic hypothesis (originating from Chiari’s original observations) viewed cerebellar tonsillar descent as a consequence of supratentorial hydrocephalus, it is now clear that this association is rare and sporadic. The tonsillar descent depending on hydrocephalus is actually transient and reversible, so that it cannot be considered a true CIM [24, 25]. Instead, the overcrowding of the foramen magnum due to CIM is considered a pathophysiological mechanism to explain the associated hydrocephalus, CIM often persisting even after a successful treatment of the concomitant hydrocephalus [48]. The main mechanism suggested by flow-sensitive phase-contrast MRI investigations would be the impairment of the CSF flow at level of the foramen magnum resulting from the compression exerted by the hindbrain herniation and the associated diseases (e.g., basilar invagination) on vallecula, cisterna magna, IV ventricle, prepontine, and premedullary cisterns [49, 50]. Such a compression was demonstrated to generate an abrupt, downward systolic displacement of brainstem and cerebellar tonsils, causing plugging and narrowing of CSF pulsations with a forced CSF transmission through the crowded foramen magnum [51]. The result could be an occlusion of the fourth ventricle outlets or a direct impairment of CSF exit from the intracranial space or both. In addition, the dissociation of CSF circulation at level of the foramen magnum would stop the pressure transmission from the pulsating vessels to the CSF, with consequent decreased CSF flow compliance in the brain (hydrocephalus) and the spinal cord (syringomyelia) [52].

These interesting hypotheses are questioned by the evidence that only a minority of CIM patients develop hydrocephalus (7–10%) [22]. Such a mismatch has been explained by some authors by postulating an occlusion of all the fourth ventricle foramina in CIM-hydrocephalic patients and a patency at least of the Luschka foramina in those without hydrocephalus [53]. It has been also postulated that the overcrowding of the foramen magnum induces a compression on the veins, which lose their ability to compensate an increased intracranial pressure [54, 55]. Accordingly, the hydrocephalus would be a condition of edema of the brain depending on a venous insufficiency [56]. The main limitation of this hypothesis remains the venous insufficiency, which is hard to be demonstrated.

**Fig. 4:**
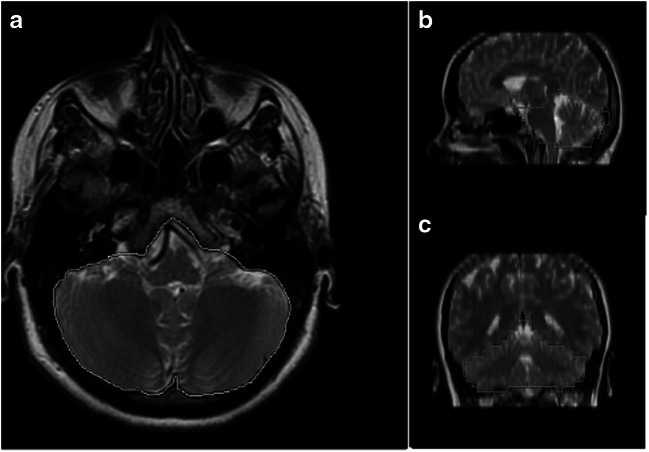
Assessment of PCF volumes by segmentation on MRI. The evaluation is realized on fast spin echo T2-weighted in the axial plane (**a**) with double check on sagittal (**b**) and coronal (**c**) views (illustrative view). The boundaries of PCF and the enclosed brain content are manually contoured on the axial images by the MRI workstation, and the resulting areas are multiplied by slice thickness plus gap and summed together to compute the total PCF volume and the PCF brain volume. The ratio between PCF brain volume and overall PCF volume is calculated to evaluate the PCF overcrowding. The PCF boundaries are delimited by clivus, pyramidal and occipital bones, tentorial hiatus, and horizontal plane including the basion and the opisthion trough the foramen magnum\

According to the only study available on this topic (Fig. 4), the PCF volumetric analysis in CIM-hydrocephalic patients does not reveal significant differences with the controls, both in children and in adults [57]. PCF overcrowding was found in adult patients as a result of the cystic IV ventricle, frequently found in adult patients compared with children [53]. These figures would explain why, in this subset of patients, the treatment of hydrocephalus (usually managed by endoscopic third ventriculostomy (ETV) is successful also in treating the symptoms related to the associated CIM. Actually, the PCF decompression after treatment of hydrocephalus is required in a minority of the cases, usually those with an evident PCF hypoplasia [22].

## Impact of PCF changes on achondroplasia

Achondroplasia is among the most frequent skeletal developmental disorders and the most common cause of dwarfism among skeletal dysplasia’s [58]. It is caused by a gain-of-function mutation in the FGFR3 gene, encoding a cellular receptor which regulates bone growth and ossification processes. The abnormal activity of the related signaling pathways leads to a diffuse alteration in the development of the skeletal system, which is responsible for the characteristic morphological features of the disease.

One of the potentially serious complications of achondroplasia is neural damage at the cervico-medullary junction due to the stenosis of the foramen magnum that, in turn, arises from the abnormal development of the occipital synchondroses (see below) typical of these patients. When clinically significant, the stenosis can lead to symptoms of spinal cord compression ranging from mild neurological disturbances to respiratory failure and death. When symptomatic, affected patients are thus candidate to foramen magnum decompression through sub-occipital craniectomy, with the aim of alleviating the impingement of the dural sac and spinal cord.

The abnormally reduced width of the foramen magnum observed in achondroplasia derives from an underdevelopment of the occipital bone components that are generated by enchondral ossification [59]. This is the cause of the shorter PCF that is found in achondroplasia together with a small foramen magnum. This reduction in PCF width seems to suggest a hypothetic link with Chiari I malformation. However, even if achondroplasia and CIM seem to share a possible common pathophysiologic mechanism (abnormal growth of the posterior fossa, which is insufficient to accommodate the growing neural structures), the consequences observed in achondroplasia are quite different and, in some ways, opposite to those typical of CIM. In particular, tonsillar herniation is only sporadically reported in achondroplasia, which is rather characterized by upward brainstem displacement [60]. The main difference between the two conditions is represented by the fact that altered values are found in almost all PCF measurements in achondroplasia, while it is not common in CIM. Actually, according to quantitative analysis of PCF in achondroplasic children, early fusion of spheno-occipital and intra-occipital synchondroses, relevant reduction of clivus and exocciput lengths, reduction of both latero-lateral and antero-posterior diameters of the foramen magnum, reduction of area of foramen magnum and jugular foramina, and, at the same time, increased length of the supraocciput, tentorial angle, cerebellar volume, PCF brain and total volume, and volume of the ventricles can be observed [61]. These features would mean that, in the context of a syndromic macrocephaly, the brain constriction in PCF is able to induce, at most, an upward cerebellar displacement but not a hindbrain herniation because of the reduced surface of the foramen magnum (which precludes the tonsillar descent).

The stenosis of the foramen magnum, which is the result of the aforementioned skull base changes and the reduced occipital interpeduncular distance, represents a main neurosurgical issue because of the risk of direct compression of the bulbo-cervical region with possible severe symptoms (hypotonia, apneas, myelopathy, sudden death). Moreover, the foramen magnum stenosis can cause abnormalities in CSF outflow from the intracranial to the spinal compartment, as evidenced with the use of MRI cine sequences [62]. In spite of that, syringomyelia is rarely observed, and the ventriculomegaly is rarely complicated by hydrocephalus in achondroplasia. This is because the ventriculomegaly is the result of the macrocephaly rather than the obstruction of the CSF pathways of the PCF. Hydrocephalus is more likely to occur when there is a concomitant stenosis of the basal synchondroses and the jugular foramina, while syringomyelia is usually found in association with cervical stenosis. The degree of venous stenosis at level of the jugular foramina well correlates with the occurrence of hydrocephalus [63]. However, in the clinical practice, both raised intracranial pressure (ICP) and syrinx are rare comorbidities [64]. On the other hand, in some children where no compression nor stenosis of the foramen magnum is noticed on neutral position, dynamic MRI can point out CSF blockage and significant compression on flexion and extension sequences, thus explaining the appearance of symptoms in this subset of patients [65].

**Fig. 5:**
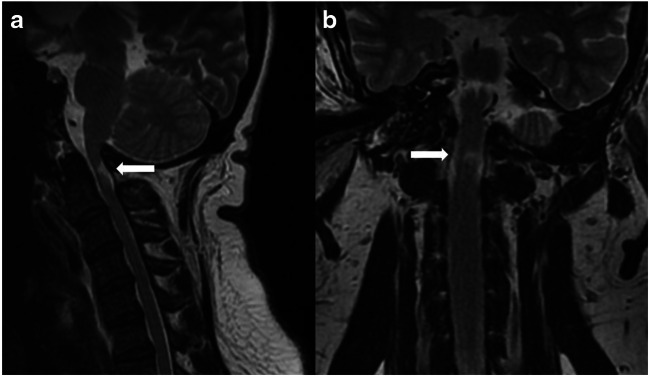
Sagittal (**a**) and coronal MRI (**b**) of the occipito-cervical region in a 28-year-old girl with achondroplasia. In spite of the mild stenosis of the foramen magnum on both antero-posterior and latero-lateral diameter, an atrophic damage of the upper cervical spinal cord can be appreciated (arrows)

Finally, the characteristic features of PCF in achondroplasia are not enough to explain the typical high-intensity intramedullary lesions of the upper cervical spinal cord found in 40–60% of adult patients [66, 67]. These lesions, usually associated with local cervical cord thinning, are thought to be the results of a focal atrophy (gliosis) (Fig. 5). However, they are found in patients without clinical signs of spinal cord compression, where also a significant stenosis of the foramen magnum can be excluded together with occipito-cervical instability. On these grounds, it has been postulated that the compression would take place during childhood as result of the disproportion between the macrocephaly and the small and weak muscles of the neck (leading to over-flexion/over-extension of the cervical spine) or as result of minimal repetitive intramedullary damages due to a reduced venous outflow [66].

## Conclusion

PCF hypoplasia is a very complex anatomical region in which every component may be affected and may lead to a different disease. A comprehensive knowledge of the matter is of extreme importance in defining the better treatment strategies for the affected patients to prevent complications and to define the prognosis.

In summary, this review showed that (1) in craniosynostoses, namely, the syndromic ones, PCF shows different degrees of hypoplasia, according to the different pattern and timing of early suture fusion. Several factors concur to PCF hypoplasia and contribute to the resulting problems (CIM, hydrocephalus), as the fusion of the major and minor sutures of the lambdoid arch, the involvement of the basal synchondroses and the occlusion of the jugular foramina. The combination of these factors explains the variety of the clinical and radiological phenotypes. (2) In primary CIM, the matter is complicated by the evidence that, in spite of impaired PCF 2D measurements and theories on the mesodermal defect, the PCF volumetry is often comparable to healthy subjects. CIM is revealed by the overcrowding of the foramen magnum that is the result of a cranio-cerebral disproportion (altered PCF brain volume/PCF total volume). Sometimes, this disproportion is evident and can be demonstrated (basilar invagination, real PCF hypoplasia); sometimes, it is not. Some recent genetic observations would suggest that CIM is the result of an excessive growth of the neural tissue rather than a reduced growth of PCF bones. (3) In achondroplasia, both macrocephaly and reduced 2D and 3D values of PCF occur. Some aspects of this disease remain partially obscure, as the rare incidence of hydrocephalus and syringomyelia and the common occurrence of asymptomatic upper cervical spinal cord damage. On the other hand, the low rate of CIM could be explained on the basis of the reduced area of the foramen magnum, which would prevent the hindbrain herniation.

## References

[1] Jeffery N (2002). Differential regional brain growth and rotation of the prenatal human tentorium cerebelli. J Anat.

[2] Cinalli G, Spennato P, Sainte-Rose C, Arnaud E, Aliberti F, Brunelle F, Cianciulli E, Renier D (2005). Chiari malformation in craniosynostosis. Childs Nerv Syst.

[3] Coll G, Abed Rabbo F, Jecko V, Sakka L, Di Rocco F, Delion M (2019). The growth of the posterior cranial fossa in FGFR2-induced faciocraniosynostosis: A review. Neurochirurgie.

[4] Coll G, Lemaire J-J, Di Rocco F, Barthélémy I, Garcier J-M, De Schlichting E, Sakka L (2016). Human foramen magnum area and posterior cranial fossa volume growth in relation to cranial base synchondrosis closure in the course of child development. Neurosurgery.

[5] Beez T, Koppel D, Sangra M (2017). Morphometric analysis of the posterior cranial fossa in syndromic and nonsyndromic craniosynostosis. J Craniofac Surg.

[6] Rehder R, Yang E, Cohen AR (2016). Variation of the slope of the tentorium during childhood. Childs Nerv Syst.

[7] Thompson DNP (2020) Chiari I malformation and associated syringomyelia. Textb. Pediatr. Neurosurg. Di Rocco C, Pang D, Rutka JT (Eds), pp 2709–2732

[8] Coll G, El Ouadih Y, Abed Rabbo F, Jecko V, Sakka L, Di Rocco F (2019). Hydrocephalus and Chiari malformation pathophysiology in FGFR2-related faciocraniosynostosis: a review. Neurochirurgie.

[9] Ghali GZ, Zaki Ghali MG, Ghali EZ, Srinivasan VM, Wagner KM, Rothermel A, Taylor J, Johnson J, Kan P, Lam S, Britz G (2019). Intracranial venous hypertension in craniosynostosis: mechanistic underpinnings and therapeutic implications. World Neurosurg.

[10] Fujisawa H, Hasegawa M, Kida S, Yamashita J (2002). A novel fibroblast growth factor receptor 2 mutation in Crouzon syndrome associated with Chiari type I malformation and syringomyelia. J Neurosurg.

[11] Richtsmeier JT (1987). Comparative study of normal, Crouzon, and Apert craniofacial morphology using finite element scaling analysis. Am J Phys Anthropol.

[12] Coll G, Arnaud E, Collet C, Brunelle F, Sainte-Rose C, Di Rocco F (2015). Skull base morphology in fibroblast growth factor receptor type 2-related faciocraniosynostosis: a descriptive analysis. Neurosurgery.

[13] Calandrelli R, D’Apolito G, Panfili M, Massimi L, Caldarelli M, Colosimo C (2016). Role of “major” and “minor” lambdoid arch sutures in posterior cranial fossa changes: mechanism of cerebellar tonsillar herniation in infants with multisutural craniosynostosis. Childs Nerv Syst.

[14] Calandrelli R, Pilato F, Massimi L, Panfili M, Colosimo C (2020). A systematic quantitative morpho-volumetric analysis in infants with sagittal craniosynostosis and relationship with the severity of scaphocephalic deformity. Radiol Med (Torino).

[15] Leikola J, Haapamäki V, Karppinen A, Koljonen V, Hukki J, Valanne L, Koivikko M (2012). Morphometric comparison of foramen magnum in non-syndromic craniosynostosis patients with or without Chiari I malformation. Acta Neurochir.

[16] Fearon JA, Dimas V, Ditthakasem K (2016). Lambdoid craniosynostosis: the relationship with Chiari deformations and an analysis of surgical outcomes. Plast Reconstr Surg.

[17] Leikola J, Koljonen V, Valanne L, Hukki J (2010). The incidence of Chiari malformation in nonsyndromic, single suture craniosynostosis. Childs Nerv Syst.

[18] Strahle J, Muraszko KM, Buchman SR, Kapurch J, Garton HJL, Maher CO (2011). Chiari malformation associated with craniosynostosis. Neurosurg Focus.

[19] Chivoret N, Arnaud E, Giraudat K, O’Brien F, Pamphile L, Meyer P, Renier D, Collet C, Di Rocco F (2018). Bilambdoid and sagittal synostosis: Report of 39 cases. Surg Neurol Int.

[20] Karppinen A, Koljonen V, Valanne L, Leikola J (2012). Asymmetric laterality of Chiari type I malformation in patients with non-syndromic single-suture craniosynostosis. Acta Neurochir.

[21] Rijken BFM, Lequin MH, van der Lijn F, van Veelen-Vincent M-LC, de Rooi J, Hoogendam YY, Niessen WJ, Mathijssen IMJ (2015). The role of the posterior fossa in developing Chiari I malformation in children with craniosynostosis syndromes. J Craniomaxillofac Surg.

[22] Massimi L, Pennisi G, Frassanito P, Tamburrini G, Di Rocco C, Caldarelli M (2019). Chiari type I and hydrocephalus. Childs Nerv Syst.

[23] Di Rocco C, Frassanito P, Massimi L, Peraio S (2011). Hydrocephalus and Chiari type I malformation. Childs Nerv Syst.

[24] Di Rocco C (2019). Should we stop using the term “malformation” for Chiari type I?. Childs Nerv Syst.

[25] Thompson DNP (2019). Chiari I-a “not so” congenital malformation?. Childs Nerv Syst.

[26] Trigylidas T, Baronia B, Vassilyadi M, Ventureyra ECG (2008). Posterior fossa dimension and volume estimates in pediatric patients with Chiari I malformations. Childs Nerv Syst.

[27] Dufton JA, Habeeb SY, Heran MKS, Mikulis DJ, Islam O (2011). Posterior fossa measurements in patients with and without Chiari I malformation. Can J Neurol Sci.

[28] Karagöz F, Izgi N, Kapíjcíjoğlu Sencer S (2002). Morphometric measurements of the cranium in patients with Chiari type I malformation and comparison with the normal population. Acta Neurochir.

[29] Marin-Padilla M, Marin-Padilla TM (1981). Morphogenesis of experimentally induced Arnold--Chiari malformation. J Neurol Sci.

[30] Nishikawa M, Sakamoto H, Hakuba A, Nakanishi N, Inoue Y (1997). Pathogenesis of Chiari malformation: a morphometric study of the posterior cranial fossa. J Neurosurg.

[31] Dagtekin A, Avci E, Kara E, Uzmansel D, Dagtekin O, Koseoglu A, Talas D, Bagdatoglu C (2011). Posterior cranial fossa morphometry in symptomatic adult Chiari I malformation patients: comparative clinical and anatomical study. Clin Neurol Neurosurg.

[32] Noudel R, Jovenin N, Eap C, Scherpereel B, Pierot L, Rousseaux P (2009). Incidence of basioccipital hypoplasia in Chiari malformation type I: comparative morphometric study of the posterior cranial fossa. Clinical article. J Neurosurg.

[33] Vurdem ÜE, Acer N, Ertekin T, Savranlar A, Inci MF (2012). Analysis of the volumes of the posterior cranial fossa, cerebellum, and herniated tonsils using the stereological methods in patients with Chiari type I malformation. ScientificWorldJournal.

[34] Roller LA, Bruce BB, Saindane AM (2015). Demographic confounders in volumetric MRI analysis: is the posterior fossa really small in the adult Chiari 1 malformation?. AJR Am J Roentgenol.

[35] Sgouros S, Kountouri M, Natarajan K (2006). Posterior fossa volume in children with Chiari malformation type I. J Neurosurg.

[36] Tubbs RS, Hill M, Loukas M, Shoja MM, Oakes WJ (2008). Volumetric analysis of the posterior cranial fossa in a family with four generations of the Chiari malformation Type I. J Neurosurg Pediatr.

[37] Sgouros S, Kountouri M, Natarajan K (2007). Skull base growth in children with Chiari malformation Type I. J Neurosurg.

[38] Capra V, Iacomino M, Accogli A, Pavanello M, Zara F, Cama A, De Marco P (2019). Chiari malformation type I: what information from the genetics?. Childs Nerv Syst.

[39] Lin GL, Hankenson KD (2011). Integration of BMP, Wnt, and notch signaling pathways in osteoblast differentiation. J Cell Biochem.

[40] Boyles AL, Enterline DS, Hammock PH, Siegel DG, Slifer SH, Mehltretter L, Gilbert JR, Hu-Lince D, Stephan D, Batzdorf U, Benzel E, Ellenbogen R, Green BA, Kula R, Menezes A, Mueller D, Oro' JJ, Iskandar BJ, George TM, Milhorat TH, Speer MC (2006). Phenotypic definition of Chiari type I malformation coupled with high-density SNP genome screen shows significant evidence for linkage to regions on chromosomes 9 and 15. Am J Med Genet A.

[41] Markunas CA, Enterline DS, Dunlap K, Soldano K, Cope H, Stajich J, Grant G, Fuchs H, Gregory SG, Ashley-Koch AE (2014). Genetic evaluation and application of posterior cranial fossa traits as endophenotypes for Chiari type I malformation. Ann Hum Genet.

[42] Musolf AM, Ho WSC, Long KA, Zhuang Z, Argersinger DP, Sun H, Moiz BA, Simpson CL, Mendelevich EG, Bogdanov EI, Bailey-Wilson JE, Heiss JD (2019). Small posterior fossa in Chiari I malformation affected families is significantly linked to 1q43-44 and 12q23-24.11 using whole exome sequencing. Eur J Hum Genet.

[43] Sadler B, Wilborn J, Antunes L, Kuensting T, Hale AT, Gannon SR, McCall K, Cruchaga C, Harms M, Voisin N, Reymond A, Cappuccio G, Brunetti-Pierri N, Tartaglia M, Niceta M, Leoni C, Zampino G, Ashley-Koch A, Urbizu A, Garrett ME, Soldano K, Macaya A, Conrad D, Strahle J, Dobbs MB, Turner TN, Shannon CN, Brockmeyer D, Limbrick DD, Gurnett CA, Haller G (2021). Rare and de novo coding variants in chromodomain genes in Chiari I malformation. Am J Hum Genet.

[44] Iskandar BJ, Quigley M, Haughton VM (2004). Foramen magnum cerebrospinal fluid flow characteristics in children with Chiari I malformation before and after craniocervical decompression. J Neurosurg.

[45] McGirt MJ, Atiba A, Attenello FJ, Wasserman BA, Datoo G, Gathinji M, Carson B, Weingart JD, Jallo GI (2008). Correlation of hindbrain CSF flow and outcome after surgical decompression for Chiari I malformation. Childs Nerv Syst.

[46] Shaffer N, Martin BA, Rocque B, Madura C, Wieben O, Iskandar BJ, Dombrowski S, Luciano M, Oshinski JN, Loth F (2014). Cerebrospinal fluid flow impedance is elevated in type I Chiari malformation. J Biomech Eng.

[47] Yan H, Han X, Jin M, Liu Z, Xie D, Sha S, Qiu Y, Zhu Z (2016). Morphometric features of posterior cranial fossa are different between Chiari I malformation with and without syringomyelia. Eur Spine J.

[48] Wen L, Ma C, Wang H, Hu Z (2014). The role of hydrocephalus in the development of Chiari I malformation and syringomyelia. J Neurol Sci.

[49] Buell TJ, Heiss JD, Oldfield EH (2015). Pathogenesis and cerebrospinal fluid hydrodynamics of the Chiari I malformation. Neurosurg Clin N Am.

[50] Hofmann E, Warmuth-Metz M, Bendszus M, Solymosi L (2000). Phase-contrast MR imaging of the cervical CSF and spinal cord: volumetric motion analysis in patients with Chiari I malformation. AJNR Am J Neuroradiol.

[51] Antonucci MU, Drohan A (2019). Dynamic cerebellar tonsils in Chiari malformation. J Pediatr.

[52] Greitz D (2004). Radiological assessment of hydrocephalus: new theories and implications for therapy. Neurosurg Rev.

[53] Decq P, Le Guérinel C, Sol JC, Brugières P, Djindjian M, Nguyen JP (2001). Chiari I malformation: a rare cause of noncommunicating hydrocephalus treated by third ventriculostomy. J Neurosurg.

[54] Häckel M, Benes V, Mohapl M (2001). Simultaneous cerebral and spinal fluid pressure recordings in surgical indications of the Chiari malformation without myelodysplasia. Acta Neurochir.

[55] Williams H (2008). The venous hypothesis of hydrocephalus. Med Hypotheses.

[56] Williams H (2008). A unifying hypothesis for hydrocephalus, Chiari malformation, syringomyelia, anencephaly and spina bifida. Cerebrospinal Fluid Res.

[57] Massimi L, Pravatà E, Tamburrini G, Gaudino S, Pettorini B, Novegno F, Colosimo C, Di Rocco C (2011). Endoscopic third ventriculostomy for the management of Chiari I and related hydrocephalus: outcome and pathogenetic implications. Neurosurgery.

[58] Pauli RM (2019). Achondroplasia: a comprehensive clinical review. Orphanet J Rare Dis.

[59] Marin-Padilla M, Marin-Padilla TM (1977). Developmental abnormalities of the occipital bone in human chondrodystrophies (achondroplasia and thanatophoric dwarfism). Birth Defects Orig Artic Ser.

[60] Nakai T, Asato R, Miki Y, Tanaka F, Matsumoto S, Konishi J (1995). A case of achondroplasia with downward displacement of the brain stem. Neuroradiology.

[61] Calandrelli R, Panfili M, D’Apolito G, Zampino G, Pedicelli A, Pilato F, Colosimo C (2017). Quantitative approach to the posterior cranial fossa and craniocervical junction in asymptomatic children with achondroplasia. Neuroradiology.

[62] Mukherjee D, Pressman BD, Krakow D, Rimoin DL, Danielpour M (2014). Dynamic cervicomedullary cord compression and alterations in cerebrospinal fluid dynamics in children with achondroplasia: review of an 11-year surgical case series. J Neurosurg Pediatr.

[63] Moritani T, Aihara T, Oguma E, Makiyama Y, Nishimoto H, Smoker WRK, Sato Y (2006). Magnetic resonance venography of achondroplasia: correlation of venous narrowing at the jugular foramen with hydrocephalus. Clin Imaging.

[64] King JAJ, Vachhrajani S, Drake JM, Rutka JT (2009). Neurosurgical implications of achondroplasia. J Neurosurg Pediatr.

[65] Danielpour M, Wilcox WR, Alanay Y, Pressman BD, Rimoin DL (2007). Dynamic cervicomedullary cord compression and alterations in cerebrospinal fluid dynamics in children with achondroplasia. Report of four cases. J Neurosurg.

[66] Brouwer PA, Lubout CM, van Dijk JM, Vleggeert-Lankamp CL (2012). Cervical high-intensity intramedullary lesions in achondroplasia: aetiology, prevalence and clinical relevance. Eur Radiol.

[67] van Dijk JMC, Lubout CMA, Brouwer PA (2007). Cervical high-intensity intramedullary lesions without spinal cord compression in achondroplasia. J Neurosurg Spine.

